# The Role of Motor Coordination, ADHD-Related Characteristics and Temperament among Mothers and Infants in Exclusive Breastfeeding: A Cohort Prospective Study

**DOI:** 10.3390/ijerph19095509

**Published:** 2022-05-01

**Authors:** Adi Freund-Azaria, Tami Bar-Shalita, Rivka Regev, Orit Bart

**Affiliations:** 1 Occupational Therapy Department, School of Health Professions, Faculty of Medicine, Tel-Aviv University, Ramat Aviv, Tel-Aviv 6997801, Israel; freundazaria@tauex.tau.ac.il (A.F.-A.); tbshalita@post.tau.ac.il (T.B.-S.); 2 Department of Neonatology, Meir Medical Center, Kfar-Saba 4428164, Israel; 3 Clalit Health Organization and Neonatal Follow-Up Clinic, Kfar-Saba 4428164, Israel; regevdr@netvision.net.il

**Keywords:** exclusive breastfeeding, breastfeeding intentions, attitudes toward breastfeeding, motor coordination, developmental coordination disorder (DCD), attention deficit hyperactivity disorder (ADHD), breastfeeding barriers, infants, mothers

## Abstract

Although exclusive breastfeeding is recommended for the first 6 months of life, breastfeeding rates are low. Motor skills and ADHD-related characteristics have not yet been examined as breastfeeding barriers. The aim of this study was to explore whether mothers’ and infants’ motor skills, mothers’ ADHD-related characteristics and infants’ temperament are associated with exclusive breastfeeding at 6 months after birth. Participants were 164 mothers and their infants recruited 2 days after birth. Mothers completed a demographic and delivery information questionnaire, the Infant Feeding Intentions Scale and the Iowa Infant Feeding Attitude Scale. At 6 months, mothers completed the Adult DCD (developmental coordination disorder)/Dyspraxia Checklist, the Adult ADHD (attention deficit hyperactivity disorder) Self-Report Scale Symptom Checklist-v1.1, and the Infant Characteristics Questionnaire, and provided information about their breastfeeding status. They were then divided into two groups accordingly: EBF (exclusive breastfeeding) and NEBF (non-exclusive breastfeeding). Infants were observed using the Test of Sensory Functions in Infants and the Alberta Infant Motor Scale. At 6 months, NEBF mothers reported higher prevalence of DCD (10.2% vs. 1.9%, χ^2^ = 5.561, *p* = 0.018) and ADHD (20.3% vs. 8.6%, χ^2^ = 4.680, *p* = 0.030) compared to EBF mothers. EBF infants demonstrated better motor coordination (t = 2.47, *p* = 0.016, *d* = 0.511), but no temperament differences compared to NEBF infants. Maternal DCD, ADHD and poor infant motor coordination are associated with non-exclusive breastfeeding and may become exclusive breastfeeding barriers. These findings may assist in identifying women at risk of not exclusively breastfeeding and encourage tailoring interventions for achieving higher exclusive breastfeeding rates.

## 1. Introduction

Exclusive breastfeeding is defined as a newborn receiving only breast milk and no other liquids or solids except for vitamins, minerals or medicines. Exclusive breastfeeding is recommended for the first 6 months of life by both the World Health Organization [1] and the American Academy of Pediatrics due to well-established benefits for mothers’ and infants’ health and for infants’ growth and development [2,3,4]. Despite these proven advantages, breastfeeding rates in most developed countries are low. For example, in the United States the rate of exclusive breastfeeding at the age of 6 months is currently only 25%, according to the Centers for Disease Control and Prevention (2020). Therefore, the Healthy People initiative has set a goal of 42% exclusive breastfeeding to be achieved by 2030 [5].

In order to succeed in increasing breastfeeding rates and to achieve the Healthy People’s goal, understanding the reasons for early cessation of breastfeeding is necessary. Indeed, studies have found correlations between exclusive breastfeeding duration and maternal factors such as: mothers’ intention to breastfeed [6,7], their attitudes toward breastfeeding [8], postnatal depression, anxiety [9,10], and the breastfeeding-related pain involved [11,12,13]. Mothers have also reported reasons for ceasing to breastfeed, such as not having enough milk [12,14,15,16], breastfeeding difficulties such as latching issues [17], their infants not gaining enough weight, medical reasons, difficulties with pumping breast milk, and the desire that others would feed the baby [11,15]. Additional factors that have emerged as related to discontinuation of breastfeeding in a qualitative study include: body image, discomfort, and difficulties and lack of confidence in breastfeeding [18]. Maternal obesity [17,19], being a single mom [15] and undergoing a C-section [6,7,16] were also found to be potential risk factors to early cessation of exclusive breastfeeding. The few studies that have examined infant-related factors to explain early cessation of breastfeeding indicate that using a pacifier and bottle feeding in the first few days might be risk factors [20,21,22]. However, to the best of our knowledge, there have been no studies examining infants’ abilities and characteristics as reasons for not being exclusively breastfed or for being breastfed for a short period of time.

To date, the examined factors, while extremely important, are not sufficient to satisfactorily explain the reasons for earlier-than-recommended cessation of breastfeeding [23,24], including when the mother wishes to continue [11]. In fact, these refer primarily to circumstantial factors and do not address the abilities and characteristics of mothers and infants required for breastfeeding activity and may affect breastfeeding duration and exclusivity. Breastfeeding is a co-occupation in which both the mother and the infant are required to be mutually responsive and physically active over time [25]. Mutual responsiveness occurs when mother and infant are reciprocally responsive to each other’s emotional tones, mainly while mother recognizes her infant’s hunger and satiety cues and readily responds. Breastfeeding, then, becomes a means for soothing and providing confidence and not only a nutritive act. Shared physical activity refers to mother and infant engaging in a reciprocal, close, linked motor activity. While the infant focuses mainly on the coordinated and efficient latching and sucking, his mother assists him in latching onto the nipple and maintaining adequate posture [26]. Thus, breastfeeding as a co-occupation consists of motor aspects and sustained attention of both mothers and infants, which have not yet been examined in this context.

Developmental coordination disorder (DCD), is characterized by an impairment in motor coordination, and attention deficit hyperactivity disorder (ADHD) is characterized by symptoms of inattention, impulsivity and hyperactivity [27]. Both diagnoses are common neurodevelopmental disorders that have a marked impact on daily activities and function throughout the lifespan [28,29,30,31]. The prevalence in the adult general population is 4.2–6.8% [32,33] and 5–6% [34] for ADHD and DCD, respectively. Although in the adult population both disorders have been extensively studied in different contexts, their impact on mothers’ propensity to breastfeed has not yet been examined.

Therefore, the aim of this study was to better understand whether and how early cessation of breastfeeding is associated with mothers’ and infants’ motor skills, mothers’ ADHD-related characteristics, and infants’ temperament. The latter includes infants’ activity level, intensity of reaction, distractibility, attention span and persistence, which may be the closest measures to reflect infants’ attention and activity characteristics at this early age [35]. We hypothesized that attitudes toward breastfeeding and breastfeeding intentions, as well as motor coordination and ADHD-related characteristics, will differ between exclusively breastfeeding (EBF) mothers and non-exclusively breastfeeding (NEBF) mothers of 6-month-old infants. We also hypothesized that 6-month-old infants of EBF and NEBF mothers will differ in their gross motor development, motor coordination and temperament.

## 2. Methods

### 2.1. Design

This study is a cohort prospective study, designed to have data collected at two time points: at 2 days and 6 months after birth, acquire relevant study factors on both infants and mothers, and follow feeding-method status at 6 months after birth. The study was conducted between June 2019 and January 2021 in a leading medical center, where the hospitalization period is 48 h after vaginal birth and 96 h after cesarean birth.

### 2.2. Sample

Mothers hospitalized at the maternity ward between June 2019 and August 2020 were recruited 2 days after birth, using a convenience sampling method. Mothers’ inclusion criteria were desire to initiate breastfeeding, more than 20 years of age, no language barriers, healthy and gave birth to a healthy single newborn between 36–42 weeks of gestation. Mothers’ exclusion criteria were undergoing chemotherapy, HIV positive, and gave birth to a newborn who needed to be fed partially or fully with a tube.

Sample size was calculated based on power analyses via G*Power 3 Software derived from *p* value of 0.05 and statistical power of 0.80. Aligned with the derived recommendations, 174 mothers and their newborn infants were recruited. Ten mothers dropped out during the first few months and did not reach the second data collection time point, resulting in a sample of 164 mothers for the final analysis. The mothers’ age ranged from 21 to 43 years (Mean (SD) 32.4 (4.2)). Infants were born between 36–42 weeks of gestation (Mean (SD) 39.0 (1.2)).

Mothers were divided into two groups according to their breastfeeding status at 6 months after birth, following the WHO definitions: (i) EBF group who exclusively breastfed [36]; specifically, in the current study EBF was limited to human milk only, mainly direct from the breast and not expressed, no complementary feeding or feeding by a wet nurse. (ii) NEBF group who did not breastfeed at all (formula feeding only) or partially breastfed (one or more formula feedings per day). The EBF group consisted of 105 mothers and their infants, and the NEBF group consisted of 59 mothers and their infants.

### 2.3. Data Collection

On the second day after birth in the maternity ward, all participating mothers provided written consent and completed self-administered paper questionnaires handed out by the main researcher. Six months after birth, participating mothers completed online self-administered questionnaires. Following the questionnaires submission, two qualified occupational therapists conducted infant assessments through home visits. In all phases of the study, the researcher collecting the data and examiners assessing the infants were blinded to the breastfeeding status and duration. The examiners were not exposed to data collected in questionnaires from the two time points. The mothers were instructed to attain for evaluation after feeding the infant and not to reveal how their infant was being fed. The examiners did not discuss with the mothers their feeding method or breastfeeding status before completing and documenting the infant’s evaluation.

### 2.4. Ethical Considerations

All aspects of the study were approved by the Institutional Ethics Review Board of the Meir Medical Center (reference number 0302-14-MMC) and by the Tel-Aviv University. Written informed consent was obtained from participating mothers, who had been assured that participation was voluntary and that they could choose to withdraw from the study at any time. Mothers’ and infants’ privacy was ensured and kept confidential.

### 2.5. Measurements

On the second day after birth in the maternity ward, participating mothers reported demographic and delivery information and completed the following questionnaires:

*The Infant Feeding Intentions Scale (IFIS)*, a standardized, reliable and valid self-report questionnaire [37], was developed to assess the strength of intention to exclusively breastfeed during the first 6 months after birth. Mothers rated how much they agree with 5 statements on a 5-point Likert scale. Total score ranges from 0 (no intention to breastfeed) to 16 (very strong intention to exclusively breastfeed). Internal consistency was demonstrated (Cronbach α = 0.9) as well as a strong significant relationship between total score and actual duration of exclusive breastfeeding [38]. Cronbach’s α for the IFIS in this study was 0.820.

*The Iowa Infant Feeding Attitude Scale (IIFAS)* [39], a standardized, reliable and valid self-report questionnaire was developed to assess maternal attitudes toward breastfeeding. Mothers rated to what extent they agree with 17 statements on a 5-point Likert scale. Total score ranges from 17 (positive formula feeding attitudes) to 85 (positive breastfeeding attitudes). The IIFAS has been demonstrated to have an internal consistency (Cronbach α > 0.8) as well as an excellent ability to predict intent to breastfeed [40]. The questionnaire Cronbach’s α in this study was 0.754.

Six months after birth, participating mothers completed the following online questionnaires:

*The Adult Developmental Coordination Disorders/Dyspraxia Checklist (ADC)* [41] is a standardized, reliable and valid self-report screening questionnaire, assessing mothers’ motor coordination. The questionnaire consists of two sections: Section A (10 questions) relates to childhood history (motor coordination experiences as a child); section B (30 questions) relates to current motor coordination functioning as an adult. Mothers rated the 40 items on a 4-point scale, describing the frequency of difficulties experienced (0 = never, 1 = sometimes, 2 = frequently, 3 = always). The total score (sections A + B) ranges from 0 to 120, where higher scores indicate more motor coordination difficulties. In addition, a participant must score at least 17 in section A and 56 or above in total in order to screen positive for DCD [42]. The ADC has been demonstrated to have an internal consistency (Cronbach α = 0.87–0.95) and is able to differentiate between a group of adults with and without DCD [41]. Cronbach’s α for the ADC in this study was 0.793, 0.889 and 0.915 for child, adult and total score, respectively.

*The Adult ADHD Self-Report Scale Symptom Checklist (ASRS-v1.1)* [43] is a standardized, reliable and valid self-report screening questionnaire used to assess symptoms of ADHD based on the 18 DSM-IV symptom criteria. This tool comprises two parts: Part A (6 questions) and part B (12 questions). For each item, mothers rated how often the stated symptom occurred over the prior 6 months, using the following rating scale: 0 = never, 1 = rarely, 2 = sometimes, 3 = often, and 4 = very often. A total score of the 18 questions ranges from 0 to 72, where higher scores indicate more ADHD-related characteristics. In addition, part A ratings of “sometimes”, “often” or “very often” on items 1–3 are assigned one point. For the remaining 4–6 items, ratings of “often” or “very often” are assigned one point. Gaining a score of 4 or more on part A is a strong indication of adult ADHD [44,45]. The ASRS has been demonstrated to have an internal consistency (Cronbach α = 0.63–0.72) and test-retest reliability (r = 0.58–0.77) as well as a strong concordance with clinician diagnoses [46]. Cronbach’s α for the ASRS continuous total scale in this study was 0.927.

*The Infant Characteristics Questionnaire (ICQ)* [47] is a standardized, reliable and valid 24-item caregiver questionnaire that assesses an infant’s temperament in 4 dimensions: ability to calm, adaptivity, activity level and ability to predict infant’s needs. Mothers rated their infant’s behavior and responses during daily routine on a 7-point scale, with the rating of 1 describing an optimal temperamental trait and 7 a difficult temperament. The ICQ has been demonstrated to have an internal consistency (Cronbach α = 0.39–0.79) and test-retest reliability (r = 0.47–0.70) as well as a convergent validity [47,48]. The questionnaire Cronbach’s α in this study was 0.771–0.892.

Additionally, at 6 months after birth, two observational tools were conducted at the homes of participating mothers and their infants.

*The Test of Sensory Functions in Infant (TSFI)*, a standardized, reliable and valid tool [49] was developed to asses sensory processing and motor coordination in infants. In this study we conducted the adaptive motor part, consisting of 5 items, which is used to evaluate motor coordination. We interpreted the scores as two categories according to the TSFI age range norm: typical (score of 7–15) vs. at risk or deficient performance (score of 0–6). The TSFI has been demonstrated to have a test-retest reliability (ICC = 0.88–0.99) and inter-rater reliability (ICC = 0.26–0.84). Content and construct validity were established [50].

*The Alberta Infant Motor Scale (AIMS)* [51] was developed to asses infant gross motor development and is a norm-referenced, observational, reliable and valid tool. It consists of 58 items at 4 different positions (prone, supine, sitting, standing). For any item observed by the examiner, 1 point is given, whereas 0 points are given when the item is not observed. The sum of all items observed gives the total raw score, ranging from 0 to 58. The total raw score is converted into a percentile rank. High percentile ranks indicate better gross motor development. The AIMS has been demonstrated to have an inter-rater reliability (ICC = 0.97–0.99), test-retest reliability (ICC = 0.85–0.99) as well as content and concurrent validity [51,52,53].

*Current Infant breastfeeding Status* (exclusive, partial or none) was reported by mothers at 6 months.

### 2.6. Data Analysis

Statistical analyses were performed with SPSS^®^ V27 (IBM Corp., Armonk, NY, USA). Data were summarized with descriptive statistics by data type. Normality for quantitative continuous variables was tested using the Shapiro–Wilk test for normality. For variables that failed to meet the normality assumption of the test, we used the common procedures to deal with distribution patterns according to their skewness (Sk) and kurtosis (K) [54,55]. The choice of the analysis scheme followed the methodological guidelines suggested by [56,57].

The sample was divided into two groups, according to their breastfeeding status at 6 months after birth: EBF and NEBF groups. Differences between groups were tested using an independent sample t-test for continuous variables, and effect size was calculated via *Cohen’s d* where values are considered small (0.2), medium (0.5) and large (0.8) [58]. Chi-square tests of independence were used to analyze binary and polytomous variables. Linear dependency between the quantitative study variables was tested using Pearson’s correlation coefficient. A 2-sided 5% level of significance was used in all hypothesis tests. Nominal *p*-values are presented.

## 3. Results

### 3.1. Analysis of Demographic and Delivery-Related Factors

No statistically significant group differences were found in the mothers’ education, family status, family income, type of delivery, and whether breastfeeding occurred in the delivery room (Table 1). Furthermore, no significant group differences were found for the mothers’ age (Mean (SD) EBF 32.1 (4.1) years vs. NEBF 33.0 (4.4) years; *t* = −1.28, *p* > 0.05). In addition, no statistically significant group differences were found in infant sex, birth order (Table 1), gestational age (Mean (SD) EBF 39.1 (1.2) vs. NEBF 38.8 (1.2); *t* = 1.84, *p* > 0.05), and birth weight (kg) (Mean (SD) EBF 3.336 (0.428) vs. NEBF 3.205 (0.369); *t* = 1.96, *p* > 0.05). As no statistically significant differences were found between EBF and NEBF, the use of covariates or confounders in the statistical analysis is redundant.

### 3.2. Analysis of Maternal Factors

Maternal factors were mostly moderately skewed: attitudes toward breastfeeding (Sk = −0.17, K = 0.42); breastfeeding intentions (Sk = −0.86, K = −0.09); motor coordination-section A (Sk = 1.6, K = 3.1); motor coordination-section B (Sk = 1.22, K = 1.60); motor coordination-total score (Sk = 1.35, K = 2.0); ADHD-related characteristics (Sk = −0.49, K = −0.05). The Shapiro–Wilk test indicated that “attitudes toward breastfeeding” was normally distributed.

Statistically significant differences between groups in attitudes toward breastfeeding were found at the first time point (i.e., on the second day after birth), demonstrating that the EBF mothers had more positive attitudes toward breastfeeding than the NEBF mothers. Furthermore, statistically significant differences between groups were found in the mothers’ scores on breastfeeding intentions at the first time point, namely the EBF group reported higher intentions to breastfeed compared to the NEBF group. We also revealed a large effect size in both attitudes and intentions (Table 2).

Maternal motor coordination was analyzed as both a continuous and binary variable (DCD/non-DCD). Statistically significant differences between groups and medium effect size were also found at the second time point (i.e., at 6 months after birth) in mothers’ motor coordination; the EBF group reported lower scores indicating better motor coordination skills, both in section B (current functioning) and in the total score (Table 2). Using the standard cut-off of >17 ADC section A items and >56 ADC total score to indicate a positive DCD screen, statistically significant differences between groups were found (χ^2^ = 5.561, *p* = 0.018), demonstrating 1.9% of mothers in the EBF group vs. 10.2% of mothers in the NEBF group screening DCD positive. These findings correspond to a 2.21 Odds Ratio (OR). Hence, for mothers with DCD, the likelihood to belong to the NEBF group 6 months after birth is 2.21 times higher than for mothers without DCD.

Similarly, “ADHD-related characteristics” was analyzed as both a continuous and binary variable (ADHD/non-ADHD). Statistically significant differences between groups as well as medium effect size were found in ADHD-related characteristics (summing both part A and part B ASRS-v1.1 items), indicating fewer ADHD-related characteristics in EBF mothers compared to NEBF mothers (Table 2). Using the standard cut-off of ≥4 ASRS-v1.1 part A items to indicate a positive ADHD screening, statistically significant differences between groups were found (χ^2^ = 4.68, *p* = 0.03) demonstrating 8.6% of mothers in the EBF group vs. 20.3% of mothers in the NEBF group screening ADHD positive. These findings correspond to a 1.74 Odds Ratio (OR). Thus, for mothers with ADHD, the likelihood to belong to the NEBF group 6 months after birth is 1.74 times higher than for mothers without ADHD.

### 3.3. Analysis of Infant Factors

Infant factors were mostly moderately skewed: motor coordination (Sk = −0.88, K = 0.07); gross motor development (Sk = −1.18, K = 1.68); temperament: *ability to calm* (Sk = 0.19, K = −0.76), *adaptivity* (Sk = 0.98, K = 0.36), *activity level* (Sk = 1.12, K = 0.57), *ability to predict infant’s needs* (Sk = 0.82, K = 0.72). The Shapiro–Wilk test indicated that *“ability to calm*” was normally distributed.

Infant motor coordination was analyzed as both a continuous and binary variable (typical/at risk or deficient). Statistically significant differences between groups and medium effect size were found in infants’ motor coordination (Table 3). Using the TSFI standard cut-off demonstrated that 12.5% of infants in the EBF group vs. 32.5% of infants in the NEBF group were found at risk or deficient motor performance (χ^2^ = 6.11, *p* = 0.013). No differences between groups were found in infants’ gross motor development, as well as in the infants’ temperament on the four subscales of the ICQ (Table 3).

### 3.4. Relations between Attitudes toward Breastfeeding and Breastfeeding Intentions, Motor Coordination and ADHD-Related Characteristics

A statistically significant strong positive correlation was found between attitudes toward breastfeeding and breastfeeding intentions (r = 0.575, *p* > 0.001). Namely, the more positive the attitudes toward breastfeeding, the greater the intentions to breastfeed exclusively and for a longer duration. Moreover, a statistically significant weak-to-moderate negative correlation was found between mothers’ attitudes toward breastfeeding and current motor coordination (r = −0.249, *p* = 0.001), and between attitudes toward breastfeeding and ADHD-related characteristics (r = −0.228, *p* = 0.003). In other words, fewer motor coordination difficulties and fewer maternal ADHD-related characteristics correlated with more positive maternal attitudes toward breastfeeding. Negative correlations were found between breastfeeding intentions and current motor coordination (r = −0.214, *p* = 0.006), and between breastfeeding intentions and ADHD-related characteristics (r = −0.185, *p* = 0.018), so that fewer motor coordination difficulties and ADHD-related characteristics correlated with greater breastfeeding intentions.

In addition, statistically significant differences were found between DCD and non-DCD mothers in attitudes toward breastfeeding and breastfeeding intentions, indicating more positive attitudes and higher breastfeeding intentions among the non-DCD mothers. Moreover, statistically significant differences were also found between ADHD and non-ADHD mothers in breastfeeding intentions, indicating higher breastfeeding intentions among the non-ADHD mothers (Table 4). We also revealed medium (*Cohen’s d* > 0.4) to large (*Cohen’s d* > 0.9) effect size in both attitudes and breastfeeding intentions for ADHD and DCD, respectively (Table 4).

## 4. Discussion

In line with our hypotheses, the mothers’ and infants’ abilities and characteristics examined in this study were associated with breastfeeding exclusivity 6 months after birth, as discussed in detail below.

### 4.1. Motor Coordination and Breastfeeding

We found differences between groups in maternal motor coordination, such that EBF mothers showed better motor coordination skills than NEBF mothers, along with lower prevalence of DCD positive screening among NEBF mothers compared to EBF mothers. This association may be due to the motor nature of breastfeeding, whereby efficient and successful breastfeeding requires a maternal bilateral motor coordination of the hands as well as eye-hand coordination. In addition, to enable the infant to latch onto the nipple, mothers are required to precisely time and coordinate their infant’s spontaneous mouth opening with their latching [26,59]. Motor coordination history, i.e., motor coordination difficulties as a child, was not found to be associated with breastfeeding exclusivity at 6 months. Despite the importance of maternal motor coordination, this is the first study, to our knowledge, to report its benefit for breastfeeding.

In examining differences between groups in the infant motor coordination at 6 months, we found that the percentage of infants in the typical range was higher in the EBF group compared to the NEBF group. On the other hand, there were no differences between groups in gross motor development. Previous studies, which have retrospectively linked infant motor development and breastfeeding, have concluded that longer exclusive breastfeeding was associated with better gross and fine motor development [60,61,62]. Conversely, other studies concluded no such association [63,64]. In our study, the findings indicate that specific difficulties in infant motor coordination, but not gross motor developmental milestones, are associated with less exclusive breastfeeding at 6 months. These findings may suggest a cause and effect whereby infant motor coordination skills may affect the establishment of effective, exclusive and prolonged breastfeeding. This is likely due to infant motor coordination skills, which encompass adequate muscle tone, postural control, and bilateral integration and endurance, having a vital role in the breastfeeding activity [26,59].

In the breastfeeding co-occupation, both mother and infant are physically active and are engaged in a reciprocally linked motor behavior [25]. Both of their motor efforts are focused on the achievement of an efficient latch onto the nipple, followed by a coordinated sucking. This needs to occur in an organized and convenient manner for both mother and infant for as long as the infant is hungry and desires to nurse [26,59]. Therefore, this co-occupation may explain our findings where mothers and infants with motor coordination difficulties experience less exclusive breastfeeding 6 months after birth compared to mothers and infants with fewer motor coordination difficulties.

### 4.2. ADHD-Related Characteristics, Temperament and Breastfeeding

Our study found that EBF mothers showed fewer ADHD-related characteristics compared to NEBF mothers, along with lower prevalence of ADHD positive screening. As of yet, studies examining the link between breastfeeding exclusivity and ADHD explored the children population solely [65,66]. Few studies suggested that children with ADHD may be at risk of not being breastfed as infants compared to children with typical development [20,67]. This current study seems to indicate that the same is true for mothers. Namely, mothers with ADHD-related characteristics may be at risk for providing their infants with a short duration of exclusive breastfeeding or for not breastfeeding at all. Since the ability to persist in exclusive breastfeeding over time requires attention, perseverance, and focus [26], mothers with more ADHD-related characteristics may lack these capabilities and accordingly may provide little or no exclusive breastfeeding to their infants.

We found no differences between groups in all four subscales of infant temperament. Although previous studies have reported associations between infant temperament and breastfeeding exclusivity and duration [35,68,69,70], our results suggest that the nature of this relationship is still unclear. The results of our study may suggest that different types of infant temperament may lead to the same breastfeeding outcome. For example, an infant with a more “calm” temperament might be very easy and satisfying to breastfeed and therefore his mother could nurse him relatively easily over time. However, a mother to an infant with a “fussy” temperament may choose to nurse as much as possible in an attempt to calm and adapt her infant into a daily routine. Therefore, the different breastfeeding outcomes may be related to other maternal behavioral factors such as their personality trait and self-efficacy [71] which should be examined in further studies.

### 4.3. Attitudes toward Breastfeeding and Breastfeeding Intentions

As expected, attitudes toward breastfeeding were associated with breastfeeding exclusivity at 6 months. In addition, significant associations were found between breastfeeding intentions and breastfeeding exclusivity. These findings are consistent with previous reports that have found that higher positive attitudes toward breastfeeding and breastfeeding intentions prior to and immediately after birth were associated with higher breastfeeding rates and breastfeeding duration [6,7,8,72]. The inter-relation between breastfeeding attitudes and breastfeeding intentions has also been demonstrated in previous studies [6,24]. However, we show novel findings with regard to maternal motor coordination and ADHD-related characteristics. Our findings suggest that mothers with more ADHD-related characteristics or mothers with a greater difficulty in motor coordination may develop more negative attitudes toward breastfeeding even before attempting to nurse. Indeed, they might perceive breastfeeding as too difficult and complex due to previous unsuccessful experiences requiring motor coordination and attention. This perception might decrease the likelihood of breastfeeding intentions and as a result, the extent of exclusive breastfeeding duration.

### 4.4. Limitations

Our study has some limitations: mothers’ motor coordination and ADHD-related characteristics were examined by self-reported questionnaires. While the subjective experience is of utmost importance, using objective assessments may broaden the understanding of the breastfeeding phenomenon. In addition, despite being a prospective study, due to the difficulty of diagnosing temperament, motor coordination and gross motor development at an early age, these assessments were obtained only at 6 months, leaving the direction of the effect not entirely clear.

## 5. Conclusions

Due to the substantial proven benefits of breastfeeding for both mothers and infants, increasing breastfeeding rates should be a major societal target. The more we explore and understand the potentially influential factors to promoting successful and sufficient breastfeeding duration, the better we will be able to address and overcome the barriers and achieve higher exclusively breastfeeding rates for 6-month-old infants. Therefore, there is a need for a continued thorough analysis of breastfeeding as a common, fundamental co-occupation of mothers and infants. Future studies should examine additional maternal and infant abilities, skills and characteristics required for exclusive and extended breastfeeding.

## Figures and Tables

**Table 1:** Mothers’ and infants’ demographic and delivery-related characteristics.

	EBF (*n* = 105)		NEBF (*n* = 59)			Total Sample (*n* = 164)
	*n*	%	*n*	%	*p*	*n* (%)
**Mothers’ education**					0.749	
High school	7	6.7	5	8.5		12 (7.3)
Higher education	7	6.7	5	8.5		12 (7.3)
University education	91	86.6	49	83		140 (85.4)
**Family status**					0.609	
Married	95	90.5	53	89.8		148 (90.2)
In a relationship	8	7.7	4	6.8		12 (7.3)
Single	1	1	2	3.4		3 (1.8)
Divorced	1	1				1 (0.7)
**Family income**					0.832	
Below Average	5	4.8	3	5.1		8 (4.9)
Average	10	9.5	4	6.8		14 (8.5)
Above Average	90	85.7	52	88.1		142 (86.6)
**Planned Pregnancy**					0.828	
Yes	92	87.6	51	86.4		143 (87.2)
No	13	12.4	8	13.6		21 (12.8)
**Pregnancy type**					0.445	
Spontaneous	98	93.3	52	88.1		150 (91.4)
With fertility treatment	4	3.8	3	5.1		7 (4.3)
With IVF	3	2.9	4	6.8		7 (4.3)
**Delivery type**					0.936	
Vaginal	79	75.2	42	71.2		121 (73.8)
Vacuum	11	10.5	7	11.9		18 (11.0)
C-Section (Epidural)	14	13.3	9	15.2		23 (14.0)
C-Section (Full anesthesia)	1	1	1	1.7		2 (1.2)
**Breastfeeding in delivery room**					0.324	
Yes	60	57.1	29	49.2		89 (54.3)
No	45	42.9	30	50.8		75 (45.7)
**Infant’s sex**					0.719	
Boy	60	57.1	32	54.2		92 (56.1)
Girl	45	42.9	27	45.8		72 (43.9)
**Birth order**					0.531	
First	46	43.8	19	32.2		65 (39.6)
Second	36	34.3	27	45.8		63 (38.4)
Third	17	16.2	10	16.9		27 (16.5)
Fourth	6	5.7	3	5.1		9 (5.5)

*Note.* Exclusively Breastfeeding (EBF), Non-Exclusively Breastfeeding (NEBF) at 6 months after birth.

**Table 2:** Differences between groups in maternal attitudes toward breastfeeding, breastfeeding intentions, motor coordination and ADHD-related characteristics.

	EBF (*n* = 105)	NEBF (*n* = 59)				Total Sample (*n* = 164)
	Mean (SD)	Mean (SD)	*t*	*p*	*Cohen’s d*	Mean (SD)
**Breastfeeding-related factors** **(Measured at 2 days)**						
Breastfeeding attitudes (IIFAS)	67.7 (6.7)	60.6 (8.4)	5.94	<0.001	0.934	65.2 (8.1)
Breastfeeding intentions (IFIS)	13.6 (2.7)	9.1 (4.2)	7.39	<0.001	1.27	11.9 (4.0)
**Motor and behavioral factors** **(Measured at 6 months)**						
Motor coordination (ADC, section A)	4.6 (4.3)	6.1 (5.6)	−1.91	0.079	0.300	5.2 (4.9)
Motor coordination (ADC, section B)	14.8 (10.4)	19.7 (12.7)	−2.49	0.014	0.422	16.6 (11.5)
Motor coordination (ADC, total score)	19.4 (13.4)	25.8 (17.7)	−2.39	0.019	0.407	21.7 (15.3)
ADHD-related characteristics (ASRS-v1.1)	21.6 (11.9)	25.7 (12.2)	−2.08	0.039	0.340	23.1 (12.2)

*Note.* Exclusively breastfeeding (EBF), Non-exclusively breastfeeding (NEBF) at 6 months after birth; Iowa Infant Feeding Attitude Scale (IIFAS); Infant Feeding Intentions Scale (IFIS); Adult Developmental Coordination Disorders/Dyspraxia Checklist (ADC) (section A-childhood history, section B-current functioning); Adult ADHD Self-Report Scale Symptom Checklist (ASRS-v1.1).

**Table 3:** Differences between groups in infant motor coordination, gross motor development and temperament at 6 months.

	EBF (*n* = 105)	NEBF (*n* = 59)				Total Sample (*n* = 164)
	Mean (SD)	Mean (SD)	*t*	*p*	*Cohen’s d*	Mean (SD)
Motor coordination (TSFI)	8.7 (1.8)	7.7 (2.1)	2.47	0.016	0.511	8.3 (2.0)
Gross motor development (AIMS)	54.6 (16.6)	55.3 (14.6)	−0.22	0.828	0.044	54.8 (15.8)
Temperament (ICQ):						
*Ability to calm*	25.4 (8.8)	23.6 (7.6)	1.32	0.190	0.218	24.8 (8.4)
*Adaptivity*	10.0 (4.2)	10.2 (4.5)	−0.30	0.763	0.045	10.1 (4.3)
*Activity level*	7.5 (3.1)	7.4 (3.3)	0.20	0.845	0.031	7.5 (3.2)
*Ability to predict infant’s needs*	10.1 (3.7)	10.9 (4.2)	−1.30	0.194	0.202	10.3 (3.9)

*Note.* Exclusively breastfeeding (EBF), Non-exclusively breastfeeding (NEBF) at 6 months after birth; Test of Sensory Functioning in Infants (TSFI, adaptive-motor subtest); Alberta Infant Motor Scale (AIMS, percentage score); Infant characteristics Questionnaire (ICQ).

**Table 4:** Differences between DCD and non-DCD mothers; ADHD and non-ADHD mothers in attitudes toward breastfeeding and breastfeeding intentions.

	DCD Mothers (*n* = 8)		Non-DCD Mothers (*n* = 155)				
	Mean	SD	Mean	SD	*t*	*p*	*Cohen’s d*
Breastfeeding attitudes (IIFAS)	58.5	5.5	65.5	8.1	2.42	0.017	1.01
Breastfeeding intentions (IFIS)	8.4	4	12.1	3.9	2.61	0.010	0.936
	**ADHD** **Mothers** **(*n* = 21)**		**Non-ADHD** **Mothers** **(*n* = 142)**				
	**Mean**	**SD**	**Mean**	**SD**	***t***	***p***	***Cohen’s d***
Breastfeeding attitudes (IIFAS)	62.4	7.3	65.6	8.1	−1.69	0.092	0.415
Breastfeeding intentions (IFIS)	10.2	4.5	12.2	3.8	−2.14	0.034	0.480

*Note.* Developmental coordination disorder (DCD); Attention deficit hyperactivity disorder (ADHD); Iowa Infant Feeding Attitude Scale (IIFAS); Infant Feeding Intentions Scale (IFIS).

## Data Availability

The data presented in this study are available on request from the corresponding author. The data are not publicity available due to participants privacy.
